# Shoulder muscle activity after latissimus dorsi transfer in an active elevation

**DOI:** 10.1016/j.jseint.2022.07.008

**Published:** 2022-08-11

**Authors:** Navin Gurnani, Derek F.P. van Deurzen, W. Jaap Willems, Thomas W.J. Janssen, DirkJan H.E.J. Veeger

**Affiliations:** a VU University, Amsterdam, the Netherlands; b OLVG, Amsterdam, the Netherlands; c DC Expert Centre, Amsterdam, the Netherlands; d Delft University of Technology, Amsterdam, the Netherlands

**Keywords:** Muscle transfer, Massive rotator cuff tear, Electromyography, Latissimus dorsi, Shoulder surgery

## Abstract

**Background:**

After latissimus dorsi transfer (LDT), an increase in scapulothoracic (ST) contribution in thoracohumeral (TH) elevation is observed when compared to the asymptomatic shoulder. It is not known which shoulder muscles contribute to this change in shoulder kinematics, and whether the timing of muscle recruitment has altered after LDT. The aim of the study was to identify which shoulder muscles and what timing of muscle recruitment are responsible for the increased ST contribution and shoulder elevation after LDT for a massive irreparable posterosuperior rotator cuff tear (MIRT).

**Methods:**

Thirteen patients with a preoperative pseudoparalysis and MIRT were recruited after LDT with a minimum follow-up of 1 year. Three-dimensional electromagnetic tracking was used to assess maximum active elevation of the shoulder (MAES) in both the LDT and the asymptomatic contralateral shoulder (ACS). Surface electromyography (EMG) tracked activation (% EMG max) and activation timing of the latissimus dorsi (LD), deltoid, teres major, trapezius (upper, middle and lower) and serratus anterior muscles were collected. MAES was studied in forward flexion, scapular abduction and abduction in the coronal plane.

**Results:**

In MAES, no difference in thoracohumeral motion was observed between the LDT and ACS, *P* = .300. However, the glenohumeral motion for MAES was significantly lower in LDT shoulders F(1,12) = 11.230, *P* = .006. The LD % EMG max did not differ between the LDT and ACS in MAES. A higher % EMG max was found for the deltoid F(1,12) = 17.241, *P* = .001, and upper trapezius F(1,10) = 13.612, *P* = .004 in the LDT shoulder during MAES. The middle trapezius only showed a higher significant difference in % EMG max for scapular abduction, *P* = .020 (LDT, 52.3 ± 19.4; ACS, 38.1 ± 19.7).The % EMG max of the lower trapezius, serratus anterior and teres major did not show any difference in all movement types between the LDT and ACS and no difference in timing of recruitment of all the shoulder muscles was observed.

**Conclusions:**

After LDT in patients with a MIRT and preoperative pseudoparalysis, the LD muscle did not alter its % EMG max during MAES when compared to the ACS. The cranial transfer of the LD tendon with its native %EMG max, together with the increased %EMG max of the deltoid, middle and upper trapezius muscles could be responsible for the increased ST contribution. The increased glenohumeral joint reaction force could in turn increase active elevation after LDT in a previous pseudoparalytic shoulder.

Shoulder kinematics change after a rotator cuff tear.^49^^,^^56^^,^^57^ In a normal shoulder, after 30 degrees of elevation, the glenohumeral (GH): scapulothoracic (ST) ratio is 2.3-2.7:1 until maximum.^4^^,^^7^ The contribution of the scapulothoracic part of the maintained thoracohumeral (TH) motion increases in patients with a cuff tear, the contribution can be restored after repair.^56^ Some posterosuperior rotator cuff tears are massive and irreparable (MIRT),^38^^,^^53^ which can be managed by muscle transfer surgery.^3^^,^^13^^,^^20^^,^^38^^,^^45^ The latissimus dorsi transfer (LDT) is a viable option described by Gerber in 1988,^17^ as it increases the active range of motion in a shoulder and reduces pain.^1^^,^^8^^,^^21^^,^^23^^,^^32^^,^^43^^,^^53^ The shoulder elevation after LDT has an increased ST contribution, which is similar to a cuff tear and does not restore kinematics to a healthy shoulder.^22^ The mode of function of the LDT is not fully understood yet. It has been postulated that the latissimus dorsi (LD) muscle changes its active function to its new mechanical role after the transfer, to elevate and externally rotate the arm.^8^^,^^15^^,^^16^^,^^23^^,^^29^^,^^32^^,^^34^ Others did not find any change of LD muscle activity in active range of motion in the shoulder, and therefore attribute the function to the tenodesis effect,^30^^,^^33^ referring to the downward directed pull of the LD on the proximal humerus, opposing the upward directed force of the deltoid in active elevation of the shoulder. This tenodesis effect might be able to create a more balanced force, acting on the GH joint, making elevation in the shoulder possible.

While the LDT can restore shoulder elevation, it does not re-establish shoulder kinematics to that of a healthy shoulder. Even after LDT, the ST contribution to TH motion in the shoulder continues to be increasing.^15^^,^^28^ Scapulothoracic muscles and recruitment timings have been analyzed in (massive) rotator cuff tears,^27^^,^^50^^,^^55^^,^^56^ subacromial impingement,^9^^,^^10^^,^^48^^,^^51^^,^^55^ glenohumeral instability,^51^ shoulder muscle fatigue,^41^^,^^52^ suprascapular nerve block,^40^ and after LDT.^29^^,^^30^ The studies reporting on muscle activity after LDT have solely focused on LD activity in its new mechanical role, whether it has changed its function from an internal rotator and adductor to an external rotator and abductor of the arm. It is not known which other shoulder muscles or changes in shoulder muscle recruitment time are responsible for the increased ST contribution in maximum active elevation of the shoulder (MAES) after LDT.

The aim of this study was to evaluate shoulder muscle activity and timing of recruitment after LDT compared to their asymptomatic contralateral shoulder (ACS) in an active elevation of the shoulder. Several muscles around the shoulder could be responsible for the increased ST contribution in MAES. Therefore, together with the muscle activity of the LD muscle, muscle activities of the scapulohumeral muscles (deltoid, teres major) and scapulothoracic muscles (trapezius and serratus anterior) were analyzed.

Our hypothesis is that the transferred LD and other scapular muscles increase their muscle activity with a difference in timing of recruitment to facilitate the increase in ST motion in MAES compared to the ACS.

## Methods

### Study design and participants

This retrospective cohort study was approved by the local medical ethical committee, OLVG (Amsterdam, the Netherlands, WO – 15.116). The patient group and mode of assessment in the present study has been reported in a prior study.^22^

Patients were recruited from 2 orthopedic clinics: OLVG (Amsterdam, NL) and Spaarne Gasthuis (Hoofddorp, NL). Participants were identified in June 2018 by searching for cases using surgical codes in the orthopedic database. Inclusion criteria were (1) LDT for a massive cuff tear (tear size >5 cm diameter with at least 2 tendons completely torn, (2) retracted and a grade 3 or higher fatty infiltration on magnetic resonance imaging),^18^^,^^36^ (3) patients with a chronic (>6 months) rotator cuff tear, failed rotator cuff repair, and/or a clinical pseudoparalysis (<90 degrees of active elevation),^12^^,^^54^ (4) no concomitant treatment of the remaining rotator cuff, (5) intact subscapularis without glenohumeral arthritis, (6) no adhesive capsulitis, (7) no previous surgery or symptoms of the contralateral shoulder, (8) no vascular or neurologic deficiencies in either arm, (9) and a follow-up of at least 1 year after LDT with an intact LD transfer on magnetic resonance imaging.

The surgical procedure had been performed as described by Gerber^17^ followed by protocolized postoperative care, which can be found in the Supplementary Appendix S1.

### 3-dimensional kinematics and MAES

The Flock-of Birds system (Ascension Technologies, Inc., Burlington, VT, USA) and accompanying software (Motion Monitor Biomech I; Innovative Sports Training, Chicago, IL, USA) was utilized for 3-dimensional kinematics. The Center for Rehabilitation and Rheumatology, Amsterdam, the Netherlands was utilized for the measurements. According to the International Society of Biomechanics standardization, proposal of the International Shoulder Group, the TH and GH motions were assessed.^24^ The highest value of the elevation angle was selected as the maximal angle for that movement and the data processed with Matlab (MathWorks, Natick, MA, USA).

MAES was analyzed by including 3 different active elevation movements as follows: forward flexion, abduction in the scapular plane, (scapular abduction) and abduction in the coronal plane. Patients were instructed to maximally move the measured arm in the respective plane, starting with the arm in neutral rotation beside the body. The measurements were repeated three times and completed at the patient’s own pace. Each shoulder was analyzed separately.

### Muscle activation

**Figure 1:**
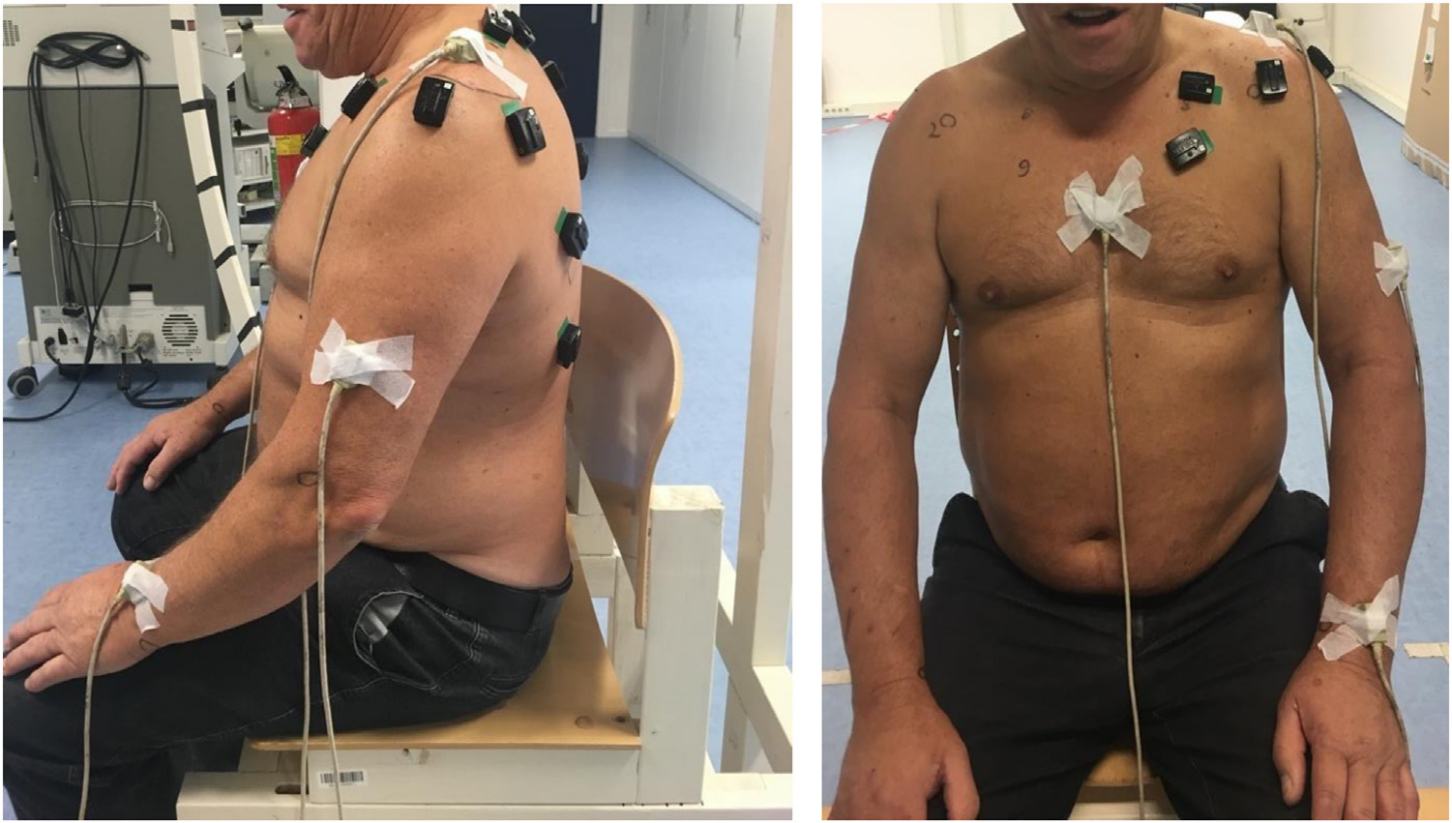
Patient setup.

Muscle activation and activation time of the LD, deltoid, teres major, serratus anterior and trapezius (upper, middle and lower) were measured with wireless EMG (Delsys Trigno Wireless, Boston, MA, USA). Location of the sensors can be viewed in Table I and patient setup in Figure 1.

**Table I:** Sensor placement electromyography.

Muscle	Sensor placement
Anterior deltoid	One finger breadth, width distal to the anterior acromion
Medial deltoid	Most lateral position on muscle
Posterior deltoid	Two finger breadths medial the angle of the acromion
Latissimus dorsi	6 cm below the angulus inferior of the scapula
Serratus Anterior	Level of the xiphoid process, lateral body contour and 45° rising to dorsal
Upper Trapezius	1/2 on the line from acromion to the spine on vertebra C7
Middle Trapezius	1/2 between the medial border of the scapula and vertebra T3
Lower Trapezius	2/3 on the line from the trigonum spinae to the vertebra T8
Teres major	Middle on the muscle belly

EMG was used to measure the activity of the muscles during active maximum shoulder elevation movements for the LDT and ACS: forward elevation, scapular abduction and abduction in coronal plane.

To scale EMG max for all muscles in the LDT and ACS, Maximal isometric voluntary contractions (MIVC) were performed in 6 different movements in a standardized order as follows: forward flexion at 45°, flexion in the scapular plane at 45°, internal and external rotation at 90°of shoulder abduction, retroflexion, amd horizontal adduction at 90° of shoulder forward flexion. Each resisted task was performed 3 times and patients had a 1-minute rest period, the largest value was used for further MIVC analysis. The researcher held and resisted the arm at the level of the wrist while the patient was asked to elevate or rotate the arm as forcefully as possible for that specific movement. Muscle activity during the MIVC was measured in millivolts (mV).

A linear envelope was achieved by correcting the Raw EMG data for offset before rectification and low-pass filtering (2Hz recursive Butterworth).

The maximal EMG value measured during the MIVCs for each muscle was used to scale the EMG signal to the maximal performance and this maximal value was set as 100% EMG max.

For further analysis, the highest EMG value during each elevation movement was selected and reported as a percentage of the EMG max of that muscle.

The timing of recruitment was reported by observing the start of the kinematic elevation curve and measuring the time to recruitment of each shoulder muscle.

### Statistical analysis

The muscle activity in % EMG max of the shoulder muscles (LD, deltoid, trapezius, serratus anterior, and teres major muscles) during MAES (forward flexion, scapular abduction, abduction in the coronal plane) for the LDT shoulder and the ACS were analyzed in a two-way repeated ANOVA with post hoc tests and Bonferroni correction. The muscle activity was also reported separately for each muscle and active shoulder elevation motion in paired T-tests. The significance level was set at 0.05.

## Results

**Figure 2:**
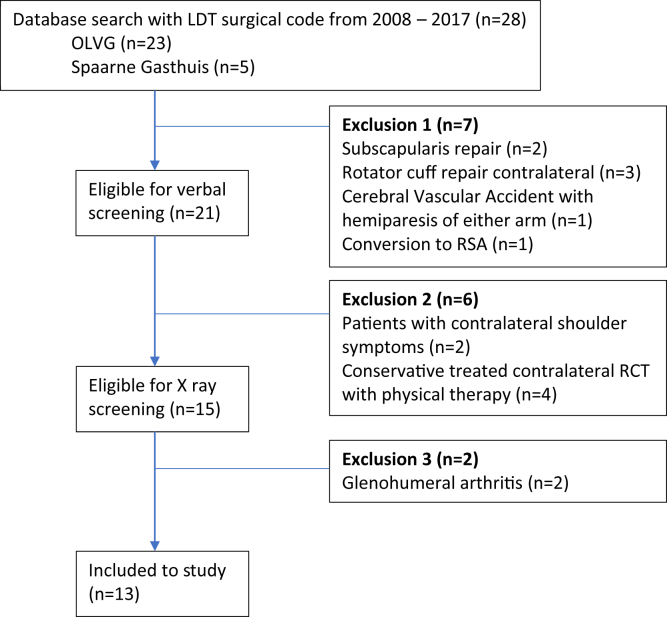
Flowchart Inclusion- and exclusion criteria.

Of the 28 eligible patients identified, 13 patients met the inclusion criteria (Fig. 2) and were included.

Patient characteristics are listed in Table II. The mean follow-up was 66.9 ± 36.7 (12-112) months. In MAES, the TH motion showed no significant difference between the LDT and ACS shoulder (F(1,12) = 1.174, *P* = .300). However, the GH motion was significantly lower in the LDT shoulder (F(1,12) = 11.230, *P* = .006). The results of the shoulder kinematics for each elevation type are reported separately and can be found in the Supplementary Appendix S1.

**Table II:** Patient characteristics.

Characteristics (N = 13)	%
Age at surgery	
60.7 years ± 3.2 (57-69)	
Gender	
Male: 10	77
Female: 3	23
Smoking perioperative	
Yes: 2	15
No: 11	85
Diabetes Mellitus	
Yes: 1	8
No: 12	92
Dominant shoulder LDT	
Yes: 8	62
No: 5	38
Body Mass Index (BMI)	
27 ± 3.2 (24.2-33.6)	
Radiology	
Hamada	
Stage I: 3	23
Stage II: 9	69
Stage III: 1	8
Posterosuperior cuff tear size	
Massive (>5 cm): 13	100
Compete tear of SSP and ISP	
Cuff tear atrophy (Goutallier)	
Grade 3: 10	76
Grade 4: 3	24
Retraction cuff tear (Patte)	
Grade 3: 13	100
Subscapularis fatty infiltration	
Grade 0: 7	54
Grade 1: 6	46
Teres minor fatty infiltration	
Grade 0: 8	61
Grade 1: 3	23
Grade 2: 1	8
Grade 3: 1	8

*LDT*, Latissimus Dorsi Transfer; *SSP*, Supraspinatus muscle; *ISP*, Infraspinatus muscle.

Muscle activity (%EMG Max, Table III).

**Table III:** Muscle activity LDT vs. ACS.

Muscle activation (% EMG max)	N	LDT	Contralateral	*P*
Latissimus dorsi				
Forward Flexion	12	11.5 ± 7.6	10.9 ± 6.9	.747
Scapular abduction	13	14.2 ± 11.1	13.3 ± 9.0	.795
Abduction	13	11.5 ± 10.4	10.4 ± 8.4	.605
Deltoid				
Forward flexion	13	73.5 ± 26.2	62.3 ± 23.9	.249
Scapular abduction	13	87.6 ± 19.4	64.0 ± 15.2	<.001
Abduction	13	88.0 ± 16.3	66.1 ± 24.9	.005
Upper Trapezius				
Forward flexion	12	45.1 ± 8.20	36.4 ± 28.6	.286
Scapular abduction	12	58.9 ± 22.0	33.7 ± 15.6	<.001
Abduction	12	60.1 ± 22.5	45.8 ± 21.0	.089
Middle Trapezius				
Forward flexion	13	39.5 ± 29.9	38.8 ± 19.9	.942
Scapular abduction	13	52.3 ± 19.4	38.1 ± 19.7	.020
Abduction	13	59.4 ± 18.9	43.9 ± 22.0	.057
Lower Trapezius				
Forward flexion	13	46.0 ± 25.5	46.8 ± 20.2	.928
Scapular abduction	13	49.6 ± 23.4	40.0 ± 12.6	.120
Abduction	13	51.7 ± 24.2	44.9 ± 23.8	.393
Serratus				
Forward flexion	11	27.7 ± 19.4	36.9 ± 12.6	.071
Scapular abduction	12	36.6 ± 27.5	31.4 ± 17.1	.348
Abduction	13	34.5 ± 27.8	34.0 ± 17.7	.939
Teres Major				
Forward flexion	12	21.0 ± 25.2	12.4 ± 5.2	.249
Scapular abduction	13	18.6 ± 23.4	11.5 ± 8.3	.337
Abduction	13	19.0 ± 24.7	9.2 ± 7.4	.200

*EMG max*, Largest electromyographic value for a specific muscle; *LDT*, Latissimus Dorsi Transfer; *ACS*, asymptomatic contralateral shoulder.

### Latissimus dorsi

In MAES, the % EMG max did not differ between the LDT and ACS shoulder, F(1,11) = 0.005, *P* = .946.

### Deltoid

The deltoid muscle had significantly higher % EMG max in MAES for the LDT shoulder, F(1,12) = 17.241, *P* = .001. When analyzing the elevation motions separately, the significant difference was seen during abduction (*P* = .005, LDT 88.0 ± 16.3, ACS 66.1 ± 24.9) and scapular abduction (*P* ≤ .001, LDT 87.6 ± 19.4, ACS 64.0 ± 15.2).

### Upper trapezius

The upper trapezius showed a higher % EMG max during MEAS for the LDT shoulder, F(1,10) = 13.612, *P* = .004. In the separate elevation motions, only scapular abduction had a significant higher % EMG max for the LDT shoulder, *P* ≤ .001 (LDT 58.9 ± 22.0, ACS 33.7 ± 15.6).

### Middle trapezius

In MAES the middle trapezius did not show any significant difference between both shoulders, F (1,12) = 3.515, *P* = .085. However, when accessing the elevation motions separately, scapular abduction showed a higher % EMG max in the LDT shoulder, *P* = .020 (LDT 52.3 ± 19.4, ACS 38.1 ± 19.7).

### Lower trapezius, serratus and teres major

No differences were found in % EMG max during MEAS between both groups.

**Figure 3:**
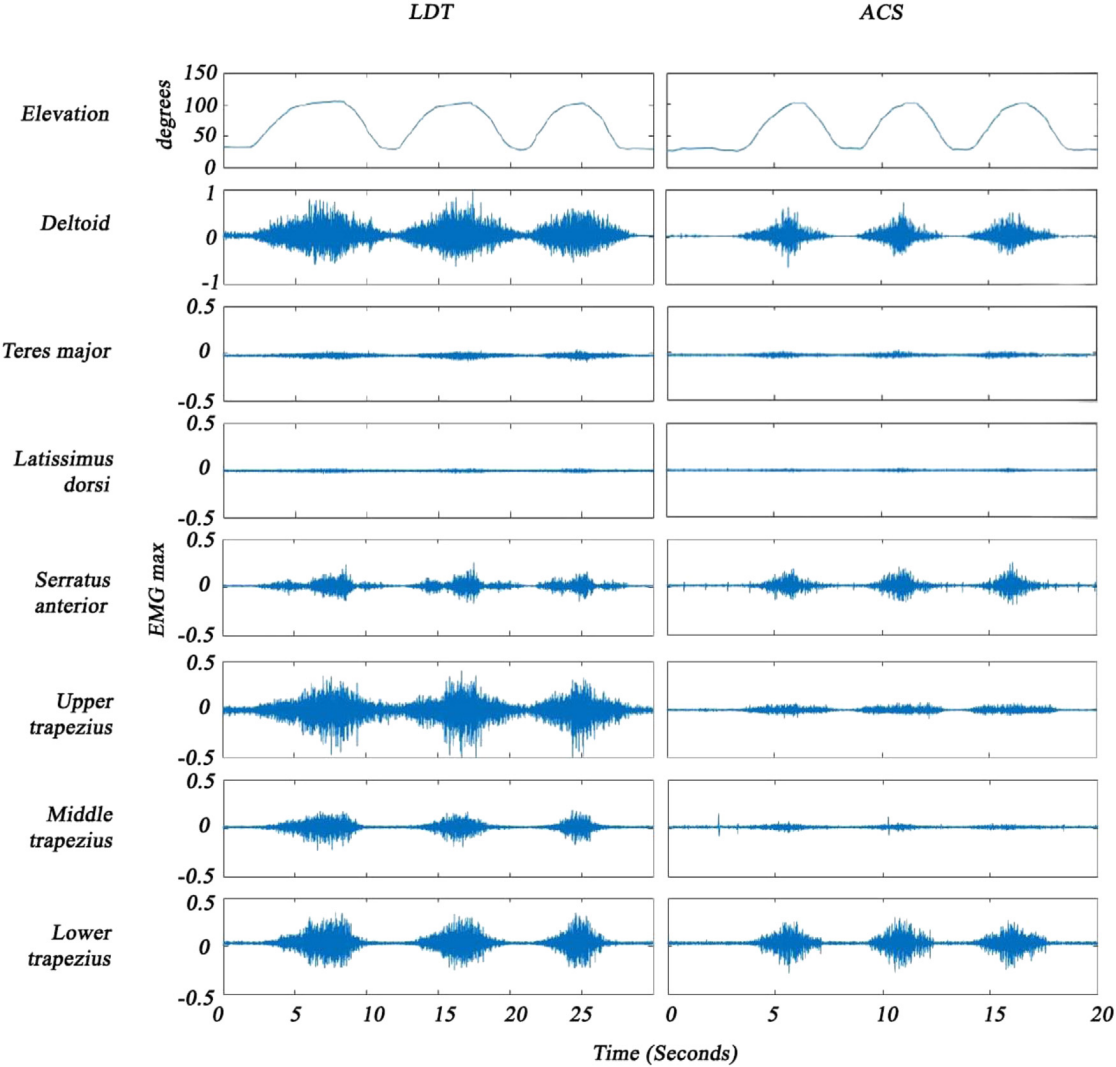
Forward flexion, muscle activity (% EMG max). *EMG max*, largest electromyographic value for a specific muscle; *LDT,* latissiumus dorsi transfer; *ACS*, asymptomatic contralateral shoulder.

**Figure 4:**
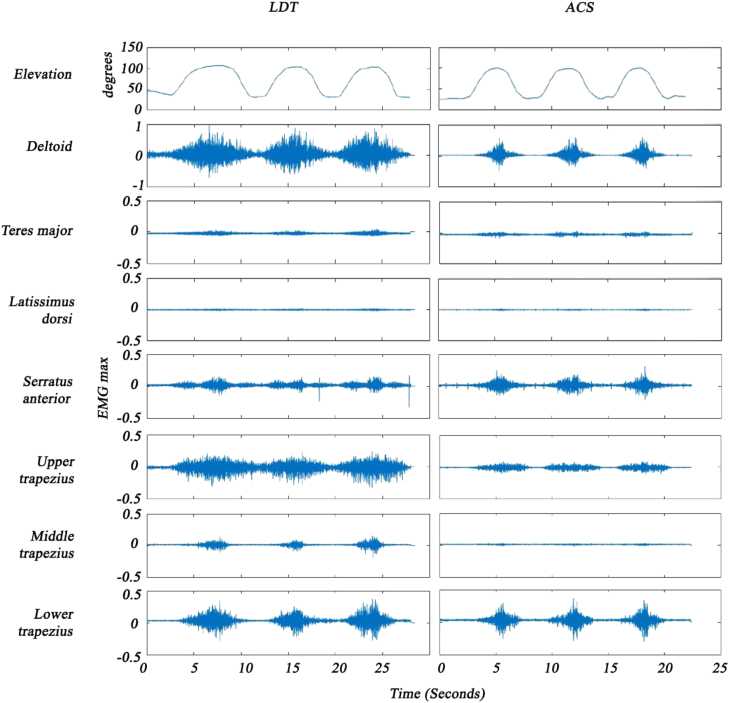
Scapular abduction, muscle activity (% EMG max). *EMG max*, largest electromyographic value for a specific muscle; *LDT*, latissiumus dorsi transfer; *ACS*, asymptomatic contralateral shoulder.

**Figure 5:**
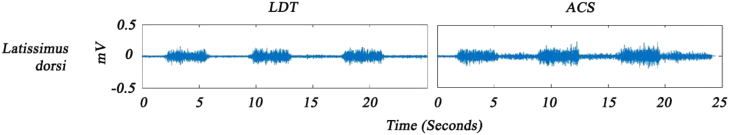
Resisted retroflexion, latissimus dorsi muscle activity (Mv). *LDT,* latissiumus dorsi transfer; *ACS*, asymptomatic contralateral shoulder.

An example of the curves for forward flexion and scapular abduction can be viewed in Figures 3 and 4, respectively. Correct placement of the EMG on the LD muscle was confirmed with resisted retroflexion (Fig. 5).

### Timing of recruitment

No difference in the recruitment time of the shoulder muscles were seen in de LDT shoulder and ACS during MEAS.

## Discussions

In our study, patients with an MIRT and preoperative pseudoparalysis had similar shoulder elevation after LDT when compared to the ACS. However, LD muscle activity was not different from its activity in the ACS, which was the case for both magnitude and timing.

The difference in muscle activity was seen in the deltoid, upper, and middle trapezius muscles.

The timing of recruitment of muscles did not differ between the LDT shoulder and ACS.

As the LD muscle did not show any difference in maximal activity after transfer compared to its ACS during MAES, our findings do not support the theory of an altered LD activity after transfer to its new mechanical role.^22^ Even more so, this study suggests that the LD muscle remains active, similar to its native role. Literature has not reached consensus with regard to the activity of the LD after transfer.^2^^,^^8^^,^^16^^,^^19^^,^^23^^,^^29^^,^^30^^,^^32^^,^^33^^,^^35^^,^^47^ One of the reasons of this inconsistency might be the method of assessment and analyzing muscle activity results. Several authors measured the MIVC of the LD to compare preoperative and postoperative muscle activity, and found an increased postoperative LD muscle activity. They attributed this finding to an active LD contraction after transfer in its new function.^23^^,^^30^^,^^31^ However, MIVC is different to isokinetic movement of the shoulder and the LD muscle increased activity could be the result of increased co-contraction after transfer seen in patients with a cuff tear.^46^^,^^49^ Others, used the contralateral LD muscle as a reference, set it to 100 % EMG max and reported EMG max values of LD in the surgical shoulder.^6^^,^^35^ However, there is a difference in muscle strength between both sides and no account for the changed EMG max after transfer is taken into consideration. In the present study we scaled the muscle activation to the MIVC of that same muscle.

Similar to our study,^22^ recent studies with analyzing isokinetic movements report no difference in LD muscle activation after LDT compared to the ACS.^15^^,^^30^

In this study, the increased activity of the deltoid muscle is seen in scapular abduction and abduction in the coronal plane after LDT, which is also reported in rotator cuff pathology, subacromial impingement, pain, and fatigue of the shoulder.^40^^,^^41^^,^^48^^,^^52^ The increase in deltoid activation after LDT in active elevation found that this study is consistent with Hetto^30^ and Henseler,^29^ which has been suggested to be necessary to compensate abduction torque from the MIRT and the additional counteracting forces of the shoulder adductors.^27^

In patients with a rotator cuff tear the shoulder adductors, ie LD, teres major and pectoralis major muscle, are more active during the active elevation.^27^ These muscles counteract the deltoid upward directed shear force during active elevation of the shoulder (co-activation), creating a stable GH fulcrum for the deltoid.^55^ After transfer, the new insertion of the LD is located more cranially and dorsally on the humeral head, contributing to better co-activation and making the downward directed pull more effective in its original muscle activation. This phenomenon might be responsible for the better-balanced forces around the GH joint in active elevation and better functional outcomes after LDT. However, we are not certain whether this is achieved actively or passively, the tenodesis effect.^30^

The strong pull of the deltoid muscle without counteracting forces could cause imbalance around the GH joint making active shoulder elevation impossible in patients with an MIRT and pseudoparalysis. It is plausible that the balance of forces around the GH joint has to be partially restored to facilitate the active elevation to overcome a pseudoparalysis of the shoulder.^49^ A possible explanation for this may be found in that the remaining rotator cuff muscles are transformed into stabilizers, increasing the GH joint reaction force and partly counteract the forces of the deltoid.^25^ This theory of change in rotator cuff function is enforced by the decreased GH motion seen after LDT.^37^^,^^39^^,^^44^ When the deltoid elevates the arm, it elevates the arm with a relatively ‘fixed’ GH joint, explaining the increase in ST contribution.

The increased ST contribution in active elevation is observed after LDT could support the LD and teres major, and to be biomechanically more effective in exerting additional GH joint reaction force to counteract the force of deltoid. A more laterally and upward rotated scapula increases the force of shoulder adductors directed to the GH joint.^5^

In this study a higher activity of the upper and middle trapezius in active scapular abduction was observed. This increase could partly be responsible for the increased ST contribution seen after LDT, a phenomenon not earlier described in literature reporting on LDT. However, studies have reported increased trapezius and serratus activity in a massive tear, or with suprascapular block simulated cuff tear.^27^^,^^40^

Shoulder muscles are recruited to create a physiological scapulothoracic rhythm in active elevation of the arm.^7^^,^^9^, ^10^, ^11^^,^^26^^,^^27^^,^^42^^,^^51^ The change in timing of activation of each shoulder muscle could also be responsible for the increased functional outcome after LDT.^51^ However, we did not find any difference in activation timing between the LDT shoulder and ACS.

### Limitations

This study has some limitations. We only were able to include 13 patients to this study; if the study is performed on a larger scale some muscle activities and different muscle activity levels could be significantly different between LDT and ACS.

The included patients did not receive any local pain inhibitor preoperative to determine, whether pain was the limiting factor of shoulder elevation. We can only assume that the LDT increased shoulder elevation.^14^

The ACS was considered to be healthy without any pathology. However, some shoulder pathologies can be asymptomatic, yet reveal different muscle activation in EMG evaluation.

In this study the MIVC for each muscle was measured in several positions for scaling the EMG max. In this assessment it was assumed that the patients truly, maximally contracted their muscle. This, however, might not always truly be happening as patients after LDT might have unconsciously held back to avoid pain. Therefore, overestimation of the activation of the muscle during the active movements may have occurred. Although the use of EMG bears the advantage of a noninvasive method of assessing muscle activity, the surface electrode attached on the skin may unintendedly have recorded the muscle activity of a different muscle. Nevertheless, the EMG data of each muscle in our study had a consistent pattern between patients.

Preoperative EMG data of the LDT shoulder was not available, making it difficult to attribute changes of % EMG max to the LDT, as it could result from a MIRT as well.

Another limitation is the large difference in follow-up time among the patients. Some patients were operated 9 years before the measurements, while for others the LD transfer was one year before. The muscles of some shoulders may, therefore, have had more time to adapt to the new situation than other muscles, possibly affecting the results of muscle activity assessment.

Future studies should investigate which muscle activation patterns of the shoulder are needed to confirm the increased downward directed force with its native activity of the LD due to the new proximal insertion site on the proximal humerus.

## Conclusions

After LDT, in MAES, the LD muscle remains active in its native form. The deltoid, upper, and middle trapeziuses increase their activity after the transfer. The combination of a more cranial insertion of the LD tendon after transfer, its native activity and the increased activity of the deltoid, middle, and lower trapezius muscles could be responsible for an increased GH joint reaction force thus, increasing active elevation in the shoulder.

## Disclaimers

Funding: No funding was disclosed by the authors.

Conflicts of interest: The authors, their immediate families, and any research foundation with which they are affiliated have not received any financial payments or other benefits from any commercial entity related to the subject of this article.

## References

[1] Anastasopoulos P.P., Alexiadis G., Spyridonos S., Fandridis E. (2017). Latissimus dorsi transfer in posterior irreparable rotator cuff tears. Open Orthop J.

[2] Aoki M., Okamura K., Fukushima S., Takahashi T., Ogino T. (1996). Transfer of latissimus dorsi for irreparable rotator-cuff tears. J Bone Joint Surg Br.

[3] Axe J.M. (2016). Tendon transfers for irreparable rotator cuff tears: an update. EFORT Open Rev.

[4] Braman J.P., Engel S.C., LaPrade R.F., Ludewig P.M. (2009). In vivo assessment of scapulohumeral rhythm during unconstrained overhead reaching in asymptomatic subjects. J Shoulder Elbow Surg.

[5] Campbell S.T., Ecklund K.J., Chu E.H., McGarry M.H., Gupta R., Lee T.Q. (2014). The role of pectoralis major and latissimus dorsi muscles in a biomechanical model of massive rotator cuff tear. J Shoulder Elbow Surg.

[6] De Casas R., Lois M., Cidoncha M., Valadron M. (2014). Clinic and electromyographic results of latissimus dorsi transfer for irreparable posterosuperior rotator cuff tears. J Orthop Surg Res.

[7] Charalambous C.P., Eastwood S. (2014). Normal and abnormal motion of the shoulder. Class Pap Orthop.

[8] Clavert P., Arndt J., Daemgen F., Kempf J.F. (2020). Long-term outcomes of latissimus dorsi transfer for irreparable rotator cuff tears. Int Orthop.

[9] Cools A.M., Declercq G.A., Cambier D.C., Mahieu N.N., Witvrouw E.E. (2007). Trapezius activity and intramuscular balance during isokinetic exercise in overhead athletes with impingement symptoms. Scand J Med Sci Sports.

[10] Cools A.M., Witvrouw E.E., Declercq G.A., Danneels L.A., Cambier D.C. (2003). Scapular muscle recruitment patterns: trapezius muscle latency with and without impingement symptoms. Am J Sports Med.

[11] Cools A.M., Witvrouw E.E., Declercq G.A., Vanderstraeten G.G., Cambier D.C. (2004). Evaluation of isokinetic force production and associated muscle activity in the scapular rotators during a protraction-retraction movement in overhead athletes with impingement symptoms. Br J Sports Med.

[12] Denard P.J., Koo S.S., Murena L., Burkhart S.S. (2012). Pseudoparalysis: the importance of rotator cable integrity. Orthopedics.

[13] Elhassan B.T., Cox R.M., Shukla D.R., Lee J., Murthi A.M., Tashjian R.Z. (2017). Management of failed rotator cuff repair in young patients. J Am Acad Orthop Surg.

[14] Ettinger L., Shapiro M., Karduna A. (2014). Subacromial injection results in further scapular dyskinesis. Orthop J Sport Med.

[15] Galasso O., Mantovani M., Muraccini M., Berardi A., De Benedetto M., Orlando N. (2020). The latissimus dorsi tendon functions as an external rotator after arthroscopic-assisted transfer for massive irreparable posterosuperior rotator cuff tears. Knee Surg Sport Traumatol Arthrosc.

[16] Gerber C. (1992). Latissimus dorsi transfer for the treatment of irreparable tears of the rotator cuff. Clin Orthop Relat Res.

[17] Gerber C., Vinh T.S., Hertel R., Hess C.W. (1988). Latissimus dorsi transfer for the treatment of massive tears of the rotator cuff. A preliminary report. Clin Orthop Relat Res.

[18] Gerber C., Wirth S.H., Farshad M. (2011). Treatment options for massive rotator cuff tears. J Shoulder Elbow Surg.

[19] Gerhardt C., Lehmann L., Lichtenberg S., Magosch P., Habermeyer P. (2010). Modified l’episcopo tendon transfers for irreparable rotator cuff tears: 5-year followup. Clin Orthop Relat Res.

[20] Greenspoon J.A., Millett P.J., Moulton S.G., Petri M. (2016). Irreparable rotator cuff tears: restoring joint kinematics by tendon transfers. Open Orthop J.

[21] Gumina S., Castricini R., De Benedetto M., Orlando N. (2016). Latissimus dorsi transfer for primary treatment of irreparable rotator cuff tears. Rotator Cuff Tear Pathog Eval Treat.

[22] Gurnani N., Willems W.J., van Deurzen D.F.P., Weening A.A., Bouwer J., Janssen T.W.J. (2022). Shoulder kinematics and muscle activity following latissimus dorsi transfer for massive irreparable posterosuperior rotator cuff tears in a pseudoparalytic shoulder. J Shoulder Elbow Surg.

[23] Habermeyer P. (2006). Transfer of the tendon of latissimus dorsi for the treatment of massive tears of the rotator cuff: a new single-incision technique. J Bone Joint Surg Br.

[24] Hamada K., Fukuda H., Mikasa M., Kobayashi Y. (1990). Roentgenographic findings in massive rotator cuff tears. A long-term observation. Clin Orthop Relat Res.

[25] Hansen M.L., Otis J.C., Johnson J.S., Cordasco F.A., Craig E.V., Warren R.F. (2008). Biomechanics of massive rotator cuff tears: implications for treatment. J Bone Joint Surg Am.

[26] Hawkes D.H., Alizadehkhaiyat O., Fisher A.C., Kemp G.J., Roebuck M.M., Frostick S.P. (2012). Normal shoulder muscular activation and co-ordination during a shoulder elevation task based on activities of daily living: an electromyographic study. J Orthop Res.

[27] Hawkes D.H., Alizadehkhaiyat O., Kemp G.J., Fisher A.C., Roebuck M.M., Frostick S.P. (2012). Shoulder muscle activation and coordination in patients with a massive rotator cuff tear: an electromyographic study. J Orthop Res.

[28] Henseler J.F., Kolk A., Zondag B., Nagels J., de Groot J.H., Nelissen R.G.H.H. (2017). Three-dimensional shoulder motion after teres major or latissimus dorsi tendon transfer for posterosuperior rotator cuff tears. J Shoulder Elbow Surg.

[29] Henseler J.F., Nagels J., Nelissen R.G.H.H., de Groot J.H. (2014). Does the latissimus dorsi tendon transfer for massive rotator cuff tears remain active postoperatively and restore active external rotation?. J Shoulder Elbow Surg.

[30] Hetto P., Spranz D., Zeifang F., Wolf S.I., van Drongelen S., Maier M.W. (2020). Muscle activity of the latissimus dorsi after tendon transfer in patients with rotator cuff tears. J Clin Med.

[31] Hodgins J.L., Kovacevic D., Purcell S., Jobin C.M., Levine W.N., Ahmad C.S. (2016). Arthroscopic suprapectoral and open subpectoral biceps tenodesis: radiographic characteristics. Arthroscopy.

[32] Iannotti J.P., Hennigan S., Herzog R., Kella S., Kelley M., Leggin B. (2006). Latissimus dorsi tendon transfer for irreparable posterosuperior rotator cuff tears: factors affecting outcome. J Bone Joint Surg Am.

[33] Ippolito G., Serrao M., Napoli F., Conte C., Miscusi M., Coppola G. (2016). Three-dimensional analysis of the shoulder motion in patients with massive irreparable cuff tears after latissimus dorsi tendon transfer (LDT). Arch Orthop Trauma Surg.

[34] Irlenbusch U., Bernsdorf M., Born S., Gansen H.K., Lorenz U. (2008). Electromyographic analysis of muscle function after latissimus dorsi tendon transfer. J Shoulder Elbow Surg.

[35] Irlenbusch U., Bracht M., Gansen H.K., Lorenz U., Thiel J. (2008). Latissimus dorsi transfer for irreparable rotator cuff tears: a longitudinal study. J Shoulder Elbow Surg.

[36] Kim J.Y., Park J.S., Rhee Y.G. (2017). Can preoperative magnetic resonance imaging predict the reparability of massive rotator cuff tears?. Am J Sports Med.

[37] Kolk A., Henseler J.F., de Witte P.B., van Zwet E.W., van der Zwaal P., Visser C.P.J. (2017). The effect of a rotator cuff tear and its size on three-dimensional shoulder motion. Clin Biomech.

[38] Kooistra B., Gurnani N., Weening A., van den Bekerom M., van Deurzen D. (2019). Low level of evidence for all treatment modalities for irreparable posterosuperior rotator cuff tears. Knee Surg Sport Traumatol Arthrosc.

[39] McCully S.P., Suprak D.N., Kosek P., Karduna A.R. (2006). Suprascapular nerve block disrupts the normal pattern of scapular kinematics. Clin Biomech.

[40] McCully S.P., Suprak D.N., Kosek P., Karduna A.R. (2007). Suprascapular nerve block results in a compensatory increase in deltoid muscle activity. J Biomech.

[41] McQuade K.J., Dawson J., Smidt G.L. (1998). Scapulothoracic muscle fatigue associated with alterations in scapulohumeral rhythm kinematics during maximum resistive shoulder elevation. J Orthop Sports Phys Ther.

[42] Moraes G.F.S., Faria C.D.C.M., Teixeira-Salmela L.F. (2008). Scapular muscle recruitment patterns and isokinetic strength ratios of the shoulder rotator muscles in individuals with and without impingement syndrome. J Shoulder Elbow Surg.

[43] Namdari S., Voleti P., Baldwin K., Glaser D., Huffman G.R. (2012). Latissimus dorsi tendon transfer for irreparable rotator cuff tears: a systematic review. J Bone Joint Surg Am.

[44] Omid R., Heckmann N., Wang L., McGarry M.H., Vangsness C.T., Lee T.Q. (2015). Biomechanical comparison between the trapezius transfer and latissimus transfer for irreparable posterosuperior rotator cuff tears. J Shoulder Elbow Surg.

[45] Omid R., Lee B. (2013). Tendon transfers for irreparable rotator cuff tears. J Am Acad Orthop Surg.

[46] Overbeek C.L., Kolk A., de Groot J.H., de Witte P.B., Gademan M.G.J., Nelissen R.G.H.H. (2019). Middle-aged adults cocontract with arm ADductors during arm ABduction, while young adults do not. Adaptations to preserve pain-free function?. J Electromyogr Kinesiol.

[47] Plath J.E., Seiberl W., Beitzel K., Minzlaff P., Schwirtz A., Imhoff A.B. (2014). Electromyographic activity after latissimus dorsi transfer: testing of coactivation as a simple tool to assess latissimus dorsi motor learning. J Shoulder Elbow Surg.

[48] Ruwe P.A., Pink M., Jobe F.W., Perry J., Scovazzo M.L. (1994). The normal and the painful shoulders during the breaststroke: electromyographic and cinematographic analysis of twelve muscles. Am J Sports Med.

[49] SAHA A.K. (1958). Zero position of the glenohumeral joint: its recognition and clinical importance. Ann R Coll Surg Engl.

[50] Steenbrink F., de Groot J.H., Veeger H.E.J., Meskers C.G.M., van de Sande M.A.J., Rozing P.M. (2006). Pathological muscle activation patterns in patients with massive rotator cuff tears, with and without subacromial anaesthetics. Man Ther.

[51] Struyf F., Cagnie B., Cools A., Baert I., Brempt J Van, Struyf P. (2014). Scapulothoracic muscle activity and recruitment timing in patients with shoulder impingement symptoms and glenohumeral instability. J Electromyogr Kinesiol.

[52] Umehara J., Kusano K., Nakamura M., Morishita K., Nishishita S., Tanaka H. (2018). Scapular kinematic and shoulder muscle activity alterations after serratus anterior muscle fatigue. J Shoulder Elbow Surg.

[53] Weening A.A., Willems W.J. (2010). Latissimus dorsi transfer for treatment of irreparable rotator cuff tears. Int Orthop.

[54] Werner C.M.L., Steinmann P.A., Gilbart M., Gerber C. (2005). Treatment of painful pseudoparesis due to irreparable rotator cuff dysfunction with the Delta III reverse-ball-and-socket total shoulder prosthesis. J Bone Joint Surg Am.

[55] de Witte P.B., Henseler J.F., Van Zwet E.W., Nagels J., Nelissen R.G.H.H., De Groot J.H. (2014). Cranial humerus translation, deltoid activation, adductor co-activation and rotator cuff disease - different patterns in rotator cuff tears, subacromial impingement and controls. Clin Biomech.

[56] de Witte P.B., van der Zwaal P., van Arkel E.R.A., Nelissen R.G.H.H., de Groot J.H. (2014). Pathologic deltoid activation in rotator cuff tear patients: normalization after cuff repair?. Med Biol Eng Comput.

[57] Yamaguchi K., Sher J.S., Andersen W.K., Garretson R., Uribe J.W., Hechtman K. (2000). Glenohumeral motion in patients with rotator cuff tears: a comparison of asymptomatic and symptomatic shoulders. J Shoulder Elbow Surg.

