# Supplementary material for: Shoulder muscle activity after latissimus dorsi transfer in an active elevation
Source: JSES Int. 2022 Aug 11;6(6):970–7. doi: 10.1016/j.jseint.2022.07.008 (PMC9637713; doi:10.1016/j.jseint.2022.07.008)
Supplement: Supplementary Appendix S1 [file mmc1.docx]

Appendix

LDT versus ACS – Shoulder elevation

| **Active movement** | **N** | **LDT** | **ACS** | **P** |
| --- | --- | --- | --- | --- |
| ROM Thoraco-humeral (°) |  |  |  |  |
| Forward flexion | 13 | 103 ± 26 | 112 ± 18 | 0.325 |
| Scapular abduction | 13 | 108 ± 25 | 115 ± 18 | 0.416 |
| Abduction | 13 | 108 ± 27 | 119 ± 17 | 0.218 |
| ROM Gleno-humeral (°) |  |  |  |  |
| Forward flexion | 13 | 57 ± 19 | 82 ± 14 | 0.004 |
| Scapular abduction | 13 | 60 ± 17 | 84 ± 19 | 0.012 |
| Abduction | 13 | 57 ± 19 | 84 ± 20 | 0.006 |

ACS: Asymptomatic contralateral shoulder

LDT: Latissimus dorsi transfer

Surgical procedure

The LDT was performed with an interscalene brachial block and general anesthesia. The LDT was performed as described by Gerber. The patient was placed in the lateral decubitus position (on the contralateral side with the affected arm on an arm rest). A 5 - 7cm S-shaped incision was made at the posterior axillary fold to harvest the LD tendon. The distal LD muscle is released from the chest wall with care of mobilizing the thoracodorsal neurovascular pedicle. The tendon is armed with two non-absorbable Ultrabraid suture (Smith & Nephew, Andover, MA, USA). An anterolateral incision of 5 cm was made to expose the interval between the anterior and middle deltoid muscles. The footprint of the supra- and infraspinatus muscle was debrided. Blunt dissection was performed to create the interval between the long head of the triceps and dorsal deltoid muscle. Two to four double loaded footprint anchors (Smith & Nephew, Andover, MA, USA) were used to fix the tendon flat covering the infraspinatus footprint and as far as possible on the supraspinatus footprint.

Postoperative care

All patients were immobilized with a shoulder sling immobilizer for 6 weeks continuously postoperatively. Active exercises of the elbow, wrist and hand were encouraged immediately after surgery, leaving the shoulder immobilized. Thereafter, passive physical therapy was started under guidance of a physical therapist. active assisted range of motion exercises were allowed from 12 weeks postoperatively arm elevation, table side and wall climb. Patients were instructed to perform exercises only within their comfort zone of tolerance and the intensity with isometric training (elevation, abduction, internal and external rotation) was increased until 6 months.
